# Idiopathic intracranial hypertension with multiple cranial nerve palsies in 10 years old thin Sudanese boy: case report

**DOI:** 10.1186/s41983-021-00339-8

**Published:** 2021-06-29

**Authors:** Mumen Abdalazim Dafallah, Elsanosi Habour, Esraa Ahmed Ragab, Zahraa Mamoun Shouk, Mohammed Izzadden

**Affiliations:** 1 Faculty of Medicine, University of Gezira, Gezira, Sudan; 2 Wad Medani Pediatric Teaching Hospital, Wad Medani, Sudan

**Keywords:** Idiopathic intracranial hypertension, Pseudotumor cerebri, Case report, Pediatrics, Sudan

## Abstract

**Background:**

Idiopathic intracranial hypertension is a rare neurological disorder of unknown etiology. It is characterized by symptoms and signs of raise intra cranial pressure, normal brain neuroimaging, and high opening pressure ≥ 280 cm H2O in the presence of normal cerebro spinal fluid constituents.

**Case presentation:**

Ten years old thin boy presented with severe throbbing headache, vomiting, and visual obscurations for a duration of 10 days. Physical examination revealed body mass index of 14.8, VI and VII cranial nerve palsies. Fudoscopy showed grade 4 papilledema; brain CT and MRI were normal. Lumbar puncture revealed pressure of 300 cm H2O with normal CSF constituents. He was treated with acetazolamide, methylprednisolone, and paracetamol.

**Conclusion:**

Pediatricians need to be more aware of idiopathic intracranial hypertension as it can lead to permanent vision loss.

## Background

Idiopathic intracranial hypertension (IIH) is a rare neurological disorder of unknown etiology. It classically affects obese female in the child-bearing age [1]. The incidence is estimated by about 0.9 cases per 100,000 in the general population [2]. Patients are usually present with signs and symptoms of raise intracranial pressure with normal cerebrospinal fluid (CSF) composition and normal brain neuroimaging [3]. The diagnosis is based on the presence of all five items of diagnostic criteria for IIH proposed by Friedman [3] which include (1) normal neurological examination except for cranial nerve abnormalities, (2) presence of papilledema, (3) normal brain neuroimaging, (4) normal CSF composition, and (5) raised lumbar puncture (LP) opening pressure (≥ 250 cm H2O in adults and ≥ 280 cm H2O in children) in a properly performed LP (Table 1).

**Table 1:** Criteria to diagnose IIH

**For a diagnosis of IIH to be made, all five criteria must be fulfilled** 1. Normal neurologic examination except for cranial nerve abnormalities (typically VI nerve/s) 2. Presence of papilledema 3. Normal brain neuroimaging (no evidence of hydrocephalus, mass or structural lesion, and no abnormal meningeal enhancement on magnetic resonance imaging). Typical radiological features of stigmata of raised intracranial pressure. 4. Normal cerebrospinal fluid composition 5. Raised lumbar puncture opening pressure (≥ 250 mmHg in adults and ≥ 280 mmHg in children (250 mmHg if the child is not sedated and not obese)) in a properly performed lumbar puncture **Diagnosis of IIH without papilledema** ➢ In the absence of papilledema, a diagnosis of IIH syndrome can be made if 2–5 from above are satisfied, and in addition the patient has unilateral or bilateral abducens nerve palsy. ➢ In the absence of papilledema or sixth nerve palsy, a diagnosis of IIH syndrome can be suggested but not made if 2–5 from above are satisfied, and in addition at least 3 of the following neuroimaging criteria are satisfied: A. Empty sella B. Flattening of the posterior aspect of the globe C. Distention of the perioptic subarachnoid space with or without a tortuous optic nerve D. Transverse venous sinus stenosis ➢ A diagnosis of IIH is definite if the patient fulfills criteria 1–5. ➢ The diagnosis is considered probable if criteria 1–4 are met but the measured CSF pressure is lower than specified for a definite diagnosis.	

We reported a unique case of IIH in a 10-year-old thin Sudanese boy with BMI of 14.8 who presented with signs and symptoms of increased intracranial pressure, VI and VII nerve palsies. The rarity of the disease in this age group made the diagnosis challenging.

## Case presentation

A 10-year-old Sudanese boy with no prior medical history, presented to our pediatric hospital, with sudden onset headache, vomiting, and visual loss for a duration of 10 days. The headache is severe, constant, throbbing in nature, aggravated by valsalva maneuver, and not associated by double vision or tinnitus. Patient also complained of vomiting several times per day mainly at early morning and has no relation to meal. Two days later, patient developed transient visual obscurations that occurred several times a day. There were no other associated symptoms. No relevant past medical history was present and patient was not on regular medications. No family history of similar condition.

**Fig. 1:**
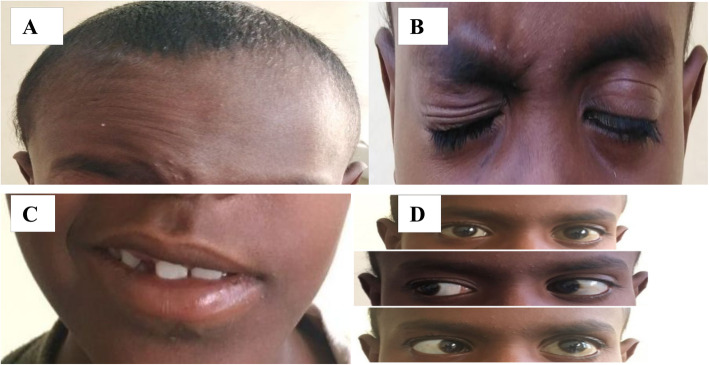
**A** Patient was unable to wrinkle forehead on the left side. **B** Unable to close left eye completely. **C** Smiling—deviation of corner of the mouth. **D** Limited abduction of the left eye (abducens nerve palsy)

Physical examination revealed thin boy with weight of 21 kg and height of 119 cm placing him below the third percentile for his age. BMI was 14.8 kg/m^2^. The patient was conscious, oriented to time, place, and person. Blood pressure 90/65 mmHg, pulse rate 110 bpm, and respiratory rate 26 c/pm. On face examination, there were obvious differences between the two sides. Reduced movement of the forehead had been noted on the left side (absent wrinkle on the left side) (Fig. 1A). Patient was unable to close his left eye completely at rest (Fig. 1B). On smiling, there was deviation of angle of the mouth and decreased activity of the muscles on the left side (Fig. 1C). No pain behind ear, no alteration in taste, no dry eyes or tearing, and patient had normal hearing. Restriction in looking at the lateral side was detected on the left side (Fig. 1D). Superior, inferior, and medial visions were normal in both eyes. So, our patient has papilledema, left facial LMNL, and left 6th nerve palsy. The remaining cranial nerves, sensory systems, reflexes, coordination, and sphincters were intact. Ophthalmological examination revealed grade 4 papilloedema. The rest of his physical and systemic examinations were normal.

**Fig. 2:**
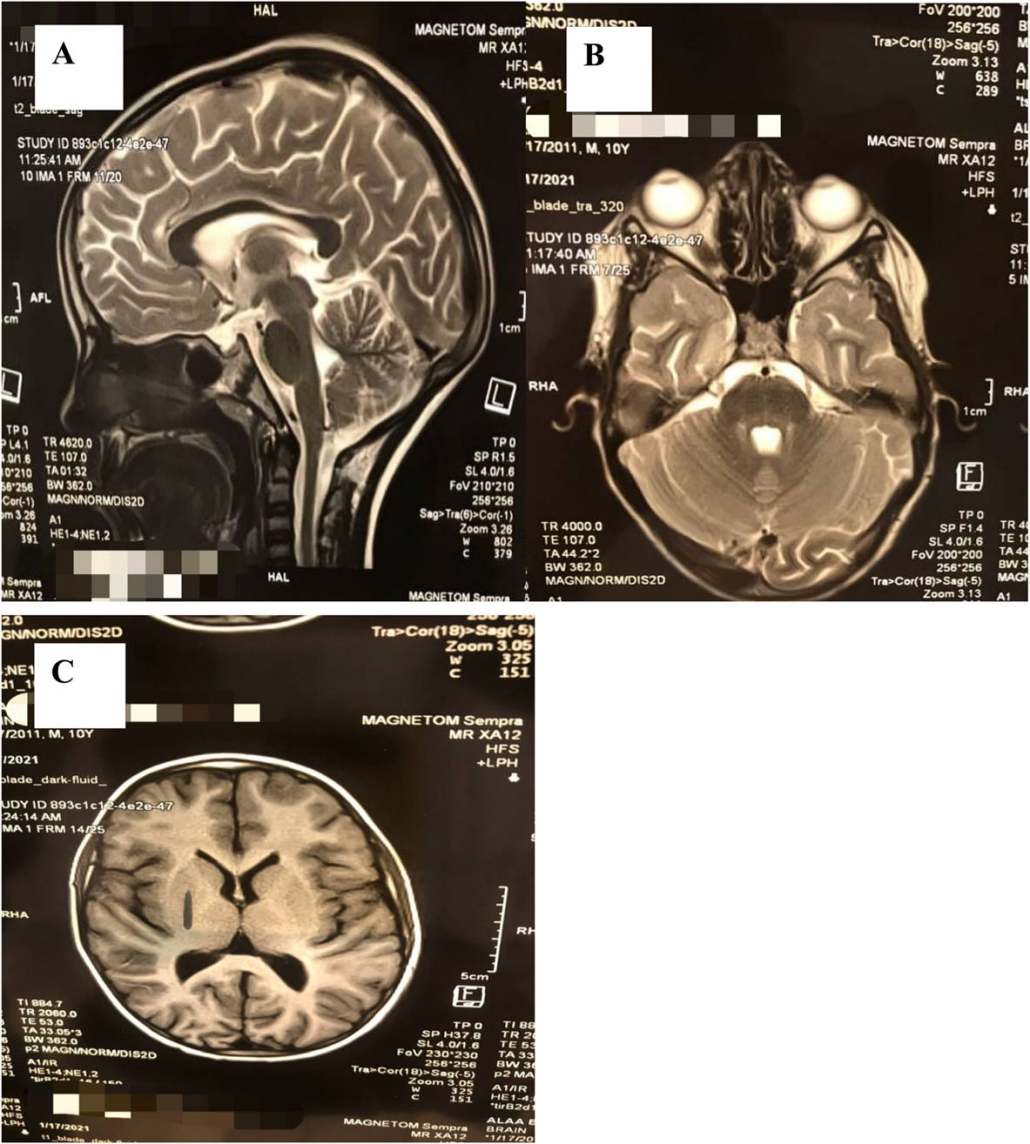
Brain MRI showed **A** normal sella turcica appearance, **B** normal perioptic disc space, and **C** normal size of ventricles

Magnetic resonance imaging (MRI) and brain computed tomography (CT) scan showed no space occupying lesion, normal sella appearance (Fig. 2A), normal perioptic disc spaces (Fig. 2B), no deformity, displacement, or obstruction of the ventricular system (Fig. 2C). Complete blood count, urine general, liver function tests, renal function tests with electrolytes, coagulation profile, thyroid function tests, and vasculitic profile were within normal ranges. LP revealed clear CSF with opening pressure of 300 cm H2O and normal CSF constituents.

The diagnosis was definite IIH as he fulfilled all items of the criteria (symptoms of increased intra cranial pressure “headache and vomiting”, no localizing findings in neurological examinations except for false localizing signs “VI nerve palsy”, normal brain CT and MRI, high opening pressure “300 cm H2O” and normal CSF constituents).

**Fig. 3:**
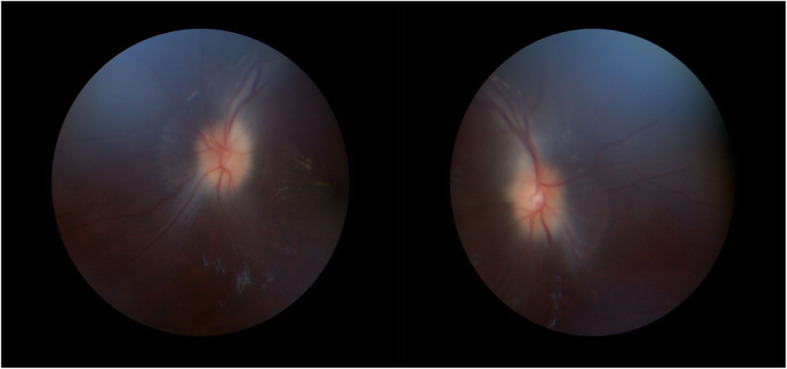
Fundoscopy showed bilateral optic disc swellings (grade 2 papilledema)

He was started on acetazolamide tablets 250 mg twice daily (25 mg/kg/day), methylprednisolone 1 mg/daily and paracetamol tablets 250 mg/6hourly. The patient clinical condition improved gradually and discharged on good condition on acetazolamide tablets 250 mg once daily. On follow-up 1 month later, there was significant improvement in headache and visual obscurations, and vomiting was stop with partial resolution of papilledema to grade 2 (Fig. 3).

### Diagnostic challenges

Magnetic resonance venography and CT venography were not done due to financial challenges. They were usually done to analyze orbit, sellar, cerebral venous sinus thrombosis, and venographic findings in IIH. Brain MRI and CT were possible alternatives which showed normal sella appearance, normal perioptic disc spaces, and normal size of ventricles.

## Discussion

Idiopathic intracranial hypertension also called pseudotumor cerebri is a rare neurological disorder in children resulting in signs and symptoms of raise intra cranial pressure in the presence of normal brain neuroimaging. The disease is first described by Quincke in 1897 who reported the first cases of IIH shortly after he discovered and introduced LP into medicine [4]. In 1904, the disease was named pseudotumor cerebri but not described until cerebral angiography was added to pneumoencephalography in 1940 [5]. Benign intracranial hypertension is another term of the disease reported by Foley in 1955 but this term “benign” is not applicable any more as the disease may cause permanent loss of vision [4, 5].

The annual incidence of pediatric IIH is about 0.5 per 100, 000 children [5]. The disease most commonly affects obese female as more than 90% of IIH patients are obese and/or in child-bearing age [6].

The commonest presentation of IIH is by headache, pulsatile tinnitus, transient visual obscurations, and loss of vision [6–8]. Children with IIH who is 10 years old or less are most commonly present with strabismus like our patient [9].

The diagnosis of IIH depends on the presence of all five items of the criteria (Table 1). Our patient was diagnosed as definite IIH as he achieved all five items of the criteria (symptoms and signs of increased intra cranial pressure, no localizing findings in neurological examinations except for false localizing signs, normal brain CT scan and MRI, and high opening pressure > 250 cm H2O with normal CSF constituents).

In order to diagnose patient as having IIH, you should first exclude secondary causes of raise intracranial pressure. Medications like tetracycline, hyper vitaminosis A, steroid thereby, lithium and oral contraceptive pills have been reported in the literature [10]. Obesity, polycystic ovary syndrome, chronic anemia, and leukemias may also be the causative factors [10, 11].

Treatment of IIH should focus on vision protection and reduce the severity of symptoms. A variety of treatments both medical and surgical can help in reducing intracranial pressure. Weight loss can induce disease remission and reduce the severity of headache and papilledema [7]. IIH is not typically associated with obesity or weight gain in pediatrics group, so weigh loss is not recommended in our patient [11].

Acetazolamide, the most common prescribing agent in IIH, inhibits carbonic anhydrase and therefore lead to decrease CSF production. Vomiting, diarrhea, renal stone, and aplastic anemia are possible side effects. The starting dose is usually 250 mg twice a day. If the patient cannot tolerate acetazolamide, toprimate, another carbonic anhydrase inhibitor, can be used in a dose of 25 mg once daily [6, 7].

Due to its UN favorable side effects, methylepredinsolone is not recommended as long-term management in IIH; however, it can be useful for short-term treatment especially in fulminant disease [12]. (Methylprednisolone may be used as a supplement to acetazolamide to hasten recovery in patients who present with severe papilledema; our patient has grade 4 papilledema. Because of their significant adverse effects, methylprednisolone was prescribed as short-term management). The usual dose of methylepredinsolone is 1 g/day [12].

Furosemide, a loop diuretic, has also been used alone or in combination with acetazolamide to reduced intracranial pressure in a low dose of 20 mg once or twice per day [11]. Other options includes subtemporal or suboccipital decompression, optic nerve sheath fenestration, CSF shunting procedures, gastric exclusion surgery, and venous sinus stenting which are not available in many resources limited hospitals.

Patients with IIH have a normal life expectancy; the major complication is papilledema which can lead to loss of vision. The most common cause of death is suicide, accidents, and medical or surgical complications [13].

## Conclusion

In conclusion, pediatric IIH is a rare neurological disorder characterize by high CSF pressure of ≥ 280 cm H2O. Blindness and loss of vision is a major concern in this disorder. Early diagnosis and treatment save patients’ vision and lead to good outcome. To the best of our knowledge, this is the first case report of pediatric IIH with multiple cranial palsies in thin Sudanese child.

## Data Availability

Not applicable

## Abbreviations

IIH: Idiopathic intracranial hypertension
CSF: Cerebrospinal fluid
cm H2O: Centimeter of water
